# A Lower Bound on the Differential Entropy of Log-Concave Random Vectors with Applications

**DOI:** 10.3390/e20030185

**Published:** 2018-03-09

**Authors:** Arnaud Marsiglietti, Victoria Kostina

**Affiliations:** 1 Center for the Mathematics of Information, California Institute of Technology, Pasadena, CA 91125, USA; 2 Department of Electrical Engineering, California Institute of Technology, Pasadena, CA 91125, USA

**Keywords:** differential entropy, reverse entropy power inequality, rate-distortion function, Shannon lower bound, channel capacity, log-concave distribution, hyperplane conjecture

## Abstract

We derive a lower bound on the differential entropy of a log-concave random variable *X* in terms of the *p*-th absolute moment of *X*. The new bound leads to a reverse entropy power inequality with an explicit constant, and to new bounds on the rate-distortion function and the channel capacity. Specifically, we study the rate-distortion function for log-concave sources and distortion measure $d\left(x,\hat{x}\right)={\left|x-\hat{x}\right|}^{r}$, with r≥1, and we establish that the difference between the rate-distortion function and the Shannon lower bound is at most $\log(\sqrt{\pi e})\approx 1.5$ bits, independently of *r* and the target distortion *d*. For mean-square error distortion, the difference is at most $\log(\sqrt{\frac{\pi e}{2}})\approx 1$ bit, regardless of *d*. We also provide bounds on the capacity of memoryless additive noise channels when the noise is log-concave. We show that the difference between the capacity of such channels and the capacity of the Gaussian channel with the same noise power is at most $\log(\sqrt{\frac{\pi e}{2}})\approx 1$ bit. Our results generalize to the case of a random vector *X* with possibly dependent coordinates. Our proof technique leverages tools from convex geometry.

## 1. Introduction

It is well known that the differential entropy among all zero-mean random variables with the same second moment is maximized by the Gaussian distribution:

(1) $$h\left(X\right)\leq \log\left(\sqrt{2\pi eE[|X{|}^{2}]}\right).$$

More generally, the differential entropy under *p*-th moment constraint is upper bounded as (see e.g., [1] (Appendix 2)), for p>0,

(2) $$\begin{array}{c} h\left(X\right)\leq \log\left(\alpha_{p}{∥X∥}_{p}\right), \end{array}$$

where

(3) $$\alpha_{p}≜2e^{\frac{1}{p}}\Gamma \left(1+\frac{1}{p}\right)p^{\frac{1}{p}},\;{∥X∥}_{p}≜{\left(E[|X{|}^{p}]\right)}^{\frac{1}{p}}.$$

Here, Γ denotes the Gamma function. Of course, if p=2, $\alpha_{p}=\sqrt{2\pi e}$, and Equation (2) reduces to Equation (1). A natural question to ask is whether a matching lower bound on h(X) can be found in terms of *p*-norm of *X*, ${∥X∥}_{p}$. The quest is meaningless without additional assumptions on the density of *X*, as h(X)=−∞ is possible even if ${∥X∥}_{p}$ is finite. In this paper, we show that if the density of *X*, $f_{X}\left(x\right)$, is *log-concave* (that is, $\log f_{X}\left(x\right)$ is concave), then h(X) stays within a constant from the upper bound in Equation (2) (see Theorem 3 in Section 2 below):

(4) $$\begin{array}{c} h\left(X\right)\geq \log\frac{{2∥X-E\left[X\right]∥}_{p}}{\Gamma {\left(p+1\right)}^{\frac{1}{p}}}, \end{array}$$

where p≥1. Moreover, the bound (4) tightens for p=2, where we have

(5) $$\begin{array}{c} h\left(X\right)\geq \frac{1}{2}\log\left(4\mathrm{Var}\left[X\right]\right). \end{array}$$

The bound (4) actually holds for p>−1 if, in addition to being log-concave, *X* is *symmetric* (that is, $f_{X}\left(x\right)=f_{X}\left(-x\right)$), (see Theorem 1 in Section 2 below).

The class of log-concave distributions is rich and contains important distributions in probability, statistics and analysis. Gaussian distribution, Laplace distribution, uniform distribution on a convex set, chi distribution are all log-concave. The class of log-concave random vectors has good behavior under natural probabilistic operations: namely, a famous result of Prékopa [2] states that sums of independent log-concave random vectors, as well as marginals of log-concave random vectors, are log-concave. Furthermore, log-concave distributions have moments of all orders.

Together with the classical bound in Equation (2), the bound in (4) tells us that entropy and moments of log-concave random variables are comparable.

Using a different proof technique, Bobkov and Madiman [3] recently showed that the differential entropy of a log-concave *X* satisfies

(6) $$h\left(X\right)\geq \frac{1}{2}\log\left(\frac{1}{2}\mathrm{Var}\left[X\right]\right).$$

Our results in (4) and (5) tighten (6), in addition to providing a comparison with other moments.

Furthermore, this paper generalizes the lower bound on differential entropy in (4) to random vectors. If the random vector $X=(X_{1},\ldots ,X_{n})$ consists of independent random variables, then the differential entropy of *X* is equal to the sum of differential entropies of the component random variables, and one can trivially apply (4) component-wise to obtain a lower bound on h(X). In this paper, we show that, even for nonindependent components, as long as the density of the random vector *X* is log-concave and satisfies a symmetry condition, its differential entropy is bounded from below in terms of covariance matrix of *X* (see Theorem 4 in Section 2 below). As noted in [4], such a generalization is related to the famous hyperplane conjecture in convex geometry. We also extend our results to a more general class of random variables, namely, the class of γ-concave random variables, with γ<0.

The bound (4) on the differential entropy allows us to derive reverse entropy power inequalities with explicit constants. The fundamental entropy power inequality of Shannon [5] and Stam [6] states that for all independent continuous random vectors *X* and *Y* in $R^{n}$,

(7) N(X+Y)≥N(X)+N(Y),

where

(8) $$N\left(X\right)=e^{\frac{2}{n}h\left(X\right)}$$

denotes the entropy power of *X*. It is of interest to characterize distributions for which a reverse form of (7) holds. In this direction, it was shown by Bobkov and Madiman [7] that, given any continuous log-concave random vectors *X* and *Y* in $R^{n}$, there exist affine volume-preserving maps $u_{1},u_{2}$ such that a reverse entropy power inequality holds for $u_{1}\left(X\right)$ and $u_{2}\left(Y\right)$:

(9) $$N\left(u_{1}\left(X\right)+u_{2}\left(Y\right)\right)\leq c\left(N\left(u_{1}\left(X\right)\right)+N\left(u_{2}\left(Y\right)\right)\right)=c\left(N\left(X\right)+N\left(Y\right)\right),$$

for some universal constant c≥1 (independent of the dimension).

In applications, it is important to know the precise value of the constant *c* that appears in (9). It was shown by Cover and Zhang [8] that, if *X* and *Y* are identically distributed (possibly dependent) log-concave random variables, then

(10) N(X+Y)≤4N(X).

Inequality (10) easily extends to random vectors (see [9]). A similar bound for the difference of i.i.d. log-concave random vectors was obtained in [10], and reads as

(11) $$N\left(X-Y\right)\leq e^{2}N\left(X\right).$$

Recently, a new form of reverse entropy power inequality was investigated in [11], and a general reverse entropy power-type inequality was developed in [12]. For further details, we refer to the survey paper [13]. In Section 5, we provide explicit constants for non-identically distributed and uncorrelated log-concave random vectors (possibly dependent). In particular, we prove that as long as log-concave random variables *X* and *Y* are uncorrelated,

(12) $$N\left(X+Y\right)\leq \frac{\pi e}{2}\left(N\left(X\right)+N\left(Y\right)\right).$$

A generalization of (12) to arbitrary dimension is stated in Theorem 8 in Section 2 below.

The bound (4) on the differential entropy is essential in the study of the difference between the rate-distortion function and the Shannon lower bound that we describe next. Given a nonnegative number *d*, the rate-distortion function $R_{X}\;\left(d\right)$ under *r*-th moment distortion measure is given by

(13) $$\begin{array}{c} R_{X}\;\left(d\right)=\underset{\begin{array}{c} P_{\hat{X}|X}:\\ E[|X-\hat{X}{|}^{r}]\leq d \end{array}}{\inf}I\left(X;\hat{X}\right), \end{array}$$

where the infimum is over all transition probability kernels R↦R satisfying the moment constraint. The celebrated Shannon lower bound [14] states that the rate-distortion function is lower bounded by

(14) $$R_{X}\;\left(d\right)\geq R_{X}\;\left(d\right)≜h\left(X\right)-\log\left(\alpha_{r}d^{\frac{1}{r}}\right),$$

where $\alpha_{r}$ is defined in (3). For mean-square distortion (r=2), (14) simplifies to

(15) $$\begin{array}{c} R_{X}\;\left(d\right)\geq h\left(X\right)-\log\sqrt{2\pi ed}. \end{array}$$

The Shannon lower bound states that the rate-distortion function is lower bounded by the difference between the differential entropy of the source and the term that increases with target distortion *d*, explicitly linking the storage requirements for *X* to the information content of *X* (measured by h(X)) and the desired reproduction distortion *d*. As shown in [15,16,17] under progressively less stringent assumptions (Koch [17] showed that (16) holds as long as H(⌊X⌋)<∞), the Shannon lower bound is tight in the limit of low distortion,

(16) $$\begin{array}{c} 0\leq R_{X}\;\left(d\right)-R_{X}\;\left(d\right)\underset{d\to 0}{\to }0. \end{array}$$

The speed of convergence in (16) and its finite blocklength refinement were recently explored in [18]. Due to its simplicity and tightness in the high resolution/low distortion limit, the Shannon lower bound can serve as a proxy for the rate-distortion function $R_{X}\;\left(d\right)$, which rarely has an explicit representation. Furthermore, the tightness of the Shannon lower bound at low *d* is linked to the optimality of simple lattice quantizers [18], an insight which has evident practical significance. Gish and Pierce [19] showed that, for mean-square error distortion, the difference between the entropy rate of a scalar quantizer, $H_{1}$, and the rate-distortion function $R_{X}\;\left(d\right)$ converges to $\frac{1}{2}\log\frac{2\pi e}{12}\approx 0.254$ bit/sample in the limit d↓0. Ziv [20] proved that $\tilde{H_{1}}-R_{X}\;\left(d\right)$ is bounded by $\frac{1}{2}\log\frac{2\pi e}{6}\approx 0.754$ bit/sample, universally in *d*, where $\tilde{H_{1}}$ is the entropy rate of a dithered scalar quantizer.

In this paper, we show that the gap between $R_{X}\;\left(d\right)$ and $R_{X}\;\left(d\right)$ is bounded universally in *d*, provided that the source density is log-concave: for mean-square error distortion (r=2 in (13)), we have

(17) $$R_{X}\;\left(d\right)-\underset{-}{R}{}_{X}\;\left(d\right)\leq \log\sqrt{\frac{\pi e}{2}}\approx 1.05\mathrm{bits}.$$

Besides leading to the reverse entropy power inequality and the reverse Shannon lower bound, the new bounds on the differential entropy allow us to bound the capacity of additive noise memoryless channels, provided that the noise follows a log-concave distribution.

The capacity of a channel that adds a memoryless noise *Z* is given by (see e.g., [21] (Chapter 9)),

(18) $$C_{Z}\left(P\right)=\underset{X:E[|X{|}^{2}]\leq P}{\sup}I\left(X;X+Z\right),$$

where *P* is the power allotted for the transmission. As a consequence of the entropy power inequality (7) (or more elementary as a consequence of the worst additive noise lemma, see [22,23]), it holds that

(19) $$C_{Z}\left(P\right)\geq \underset{-}{C}{}_{Z}\left(P\right)=\frac{1}{2}\log\left(1+\frac{P}{\mathrm{Var}[Z]}\right),$$

for arbitrary noise *Z*, where $\underset{-}{C}{}_{Z}\left(P\right)$ denotes the capacity of the additive white Gaussian noise channel with noise variance Var[Z]. This fact is well known (see e.g., [21] (Chapter 9)), and is referred to as the saddle-point condition.

In this paper, we show that, whenever the noise *Z* is log-concave, the difference between the capacity $C_{Z}\left(P\right)$ and the capacity of a Gaussian channel with the same noise power satisfies

(20) $$C_{Z}\left(P\right)-C_{Z}\left(P\right)\leq \log\sqrt{\frac{\pi e}{2}}\approx 1.05\mathrm{bits}.$$

Let us mention a similar result by Zamir and Erez [24], who showed that the capacity of an arbitrary memoryless additive noise channel is well approximated by the mutual information between the Gaussian input and the output of the channel:

(21) $$C_{Z}\left(P\right)-I\left(X^{*};X^{*}+Z\right)\leq \frac{1}{2}\mathrm{bits},$$

where $X^{*}$ is a Gaussian input satisfying the power constraint. The bounds (20) and (21) are not directly comparable.

The rest of the paper is organized as follows. Section 2 presents and discusses our main results: the lower bounds on differential entropy in Theorems 1, 3 and 4, the reverse entropy power inequalities with explicit constants in Theorems 7 and 8, the upper bounds on $R_{X}\;\left(d\right)-{\underline{R}}_{X}\;\left(d\right)$ in Theorems 9 and 10, and the bounds on the capacity of memoryless additive channels in Theorems 12 and 13. The convex geometry tools served to prove the bounds on differential entropy and the bounds in Theorems 1, 3 and 4 are presented in Section 3. In Section 4, we extend our results to the class of γ-concave random variables. The reverse entropy power inequalities in Theorems 7 and 8 are proven in Section 5. The bounds on the rate-distortion function in Theorems 9 and 10 are proven in Section 6. The bounds on the channel capacity in Theorems 12 and 13 are proven in Section 7.

## 2. Main Results

### 2.1. Lower Bounds on the Differential Entropy

A function $f:R^{n}\to \left[0,+\infty \right)$ is *log-concave* if $\log f:R^{n}\to \left[-\infty ,\infty \right)$ is a concave function. Equivalently, *f* is log-concave if for every λ∈[0,1] and for every $x,y\in R^{n}$, one has

(22) $$\begin{array}{c} f\left(\left(1-\lambda \right)x+\lambda y\right)\geq f{\left(x\right)}^{1-\lambda }f{\left(y\right)}^{\lambda }. \end{array}$$

We say that a random vector *X* in $R^{n}$ is log-concave if it has a probability density function $f_{X}$ with respect to Lebesgue measure in $R^{n}$ such that $f_{X}$ is log-concave.

Our first result is a lower bound on the differential entropy of symmetric log-concave random variable in terms of its moments.

**Theorem** **1.** *Let X be a symmetric log-concave random variable. Then, for every p>−1,*

(23) $$\begin{array}{c} h\left(X\right)\geq \log\frac{{2∥X∥}_{p}}{\Gamma {\left(p+1\right)}^{\frac{1}{p}}}. \end{array}$$

*Moreover, *(23)* holds with equality for uniform distribution in the limit p↓−1.*

As we will see in Theorem 3, for p=2, the bound (23) tightens as

(24) $$\begin{array}{c} h\left(X\right)\geq \log(2∥X{∥}_{2}). \end{array}$$

The difference between the upper bound in (2) and the lower bound in (23) grows as log(*p*) as p→+∞, as $\frac{1}{\sqrt{p}}$ as $p\to 0^{+}$, and reaches its minimum value of log(*e*) ≈ 1.4 bits at p=1.

The next theorem, due to Karlin, Proschan and Barlow [25], shows that the moments of a symmetric log-concave random variable are comparable, and demonstrates that the bound in Theorem 1 tightens as p↓−1.

**Theorem** **2.** *Let X be a symmetric log-concave random variable. Then, for every −1<p≤q,*

(25) $$\begin{array}{c} \frac{{∥X∥}_{q}}{\Gamma {\left(q+1\right)}^{\frac{1}{q}}}\leq \frac{{∥X∥}_{p}}{\Gamma {\left(p+1\right)}^{\frac{1}{p}}}. \end{array}$$

*Moreover, the Laplace distribution satisfies *(25)* with equality [25].*

Combining Theorem 2 with the well-known fact that ${∥X∥}_{p}$ is non-decreasing in *p*, we deduce that for every symmetric log-concave random variable *X*, for every −1<p<q,

(26) $$\begin{array}{c} {∥X∥}_{p}\leq {∥X∥}_{q}\leq \frac{\Gamma {\left(q+1\right)}^{\frac{1}{q}}}{\Gamma {\left(p+1\right)}^{\frac{1}{p}}}{∥X∥}_{p}. \end{array}$$

Using Theorem 1 and (24), we immediately obtain the following upper bound for the relative entropy $D(X||G_{X})$ between a symmetric log-concave random variable *X* and a Gaussian $G_{X}$ with same variance as that of *X*.

**Corollary** **1.** *Let X be a symmetric log-concave random variable. Then, for every p>−1,*

(27) $$\begin{array}{c} D(X||G_{X})\leq \log\sqrt{\pi e}+\Delta_{p}, \end{array}$$

*where $G_{X}∼{N(0,∥X∥}_{2}^{2})$, and*

(28) $$\Delta_{p}≜\left\{\begin{array}{cc} \log\left(\frac{\Gamma {\left(p+1\right)}^{\frac{1}{p}}}{\sqrt{2}}\frac{{∥X∥}_{2}}{{∥X∥}_{p}}\right), & p\neq 2,\\ -\log\sqrt{2}, & p=2. \end{array}\right.$$

**Remark** **1.** *The uniform distribution achieves equality in *(27)* in the limit* p↓−1. *Indeed, if U is uniformly distributed on a symmetric interval, then*

(29) $$\Delta_{p}=\log\frac{\Gamma {\left(p+2\right)}^{\frac{1}{p}}}{\sqrt{6}}\underset{p\to -1}{\to }\frac{1}{2}\log\frac{1}{6},$$

*and so, in the limit p↓−1, the upper bound in Corollary 1 coincides with the true value of $D(U||G_{U})$:*

(30) $$\begin{array}{c} D(U||G_{U})=\frac{1}{2}\log\frac{\pi e}{6}. \end{array}$$

We next provide a lower bound for the differential entropy of log-concave random variables that are not necessarily symmetric.

**Theorem** **3.** *Let X be a log-concave random variable. Then, for every p≥1,*

(31) $$\begin{array}{c} h\left(X\right)\geq \log\frac{{2∥X-E\left[X\right]∥}_{p}}{\Gamma {\left(p+1\right)}^{\frac{1}{p}}}. \end{array}$$

*Moreover, for p=2, the bound *(31)* tightens as*

(32) $$h\left(X\right)\geq \log\left(2\sqrt{\mathrm{Var}[X]}\right).$$

The next proposition is an analog of Theorem 2 for log-concave random variables that are not necessarily symmetric.

**Proposition** **1.** *Let X be a log-concave random variable. Then, for every 1≤p≤q,*

(33) $$\frac{{∥X-E\left[X\right]∥}_{q}}{\Gamma {\left(q+1\right)}^{\frac{1}{q}}}\leq 2\frac{{∥X-E\left[X\right]∥}_{p}}{\Gamma {\left(p+1\right)}^{\frac{1}{p}}}.$$

**Remark** **2.** Contrary to Theorem 2, we do not know whether there exists a distribution that realizes equality in *(33)*.

Using Theorem 3, we immediately obtain the following upper bound for the relative entropy $D(X||G_{X})$ between an arbitrary log-concave random variable *X* and a Gaussian $G_{X}$ with same variance as that of *X*. Recall the definition of $\Delta_{p}$ in (28).

**Corollary** **2.** *Let X be a zero-mean, log-concave random variable. Then, for every p≥1,*

(34) $$\begin{array}{c} D(X||G_{X})\leq \log\sqrt{\pi e}+\Delta_{p}, \end{array}$$

*where $G_{X}∼{N(0,∥X∥}_{2}^{2})$. In particular, by taking p=2, we necessarily have*

(35) $$\begin{array}{c} D(X||G_{X})\leq \log\sqrt{\frac{\pi e}{2}}. \end{array}$$

For a given distribution of *X*, one can optimize over *p* to further tighten (35), as seen in (29) for the uniform distribution.

We now present a generalization of the bound in Theorem 1 to random vectors satisfying a symmetry condition. A function $f:R^{n}\to R$ is called *unconditional* if, for every $\left(x_{1},\cdots ,x_{n}\right)\in R^{n}$ and every $\left(\varepsilon_{1},\cdots ,\varepsilon_{n}\right)\in {\left\{-1,1\right\}}^{n}$, one has

(36) $$\begin{array}{c} f\left(\varepsilon_{1}x_{1},\cdots ,\varepsilon_{n}x_{n}\right)=f\left(x_{1},\cdots ,x_{n}\right). \end{array}$$

For example, the probability density function of the standard Gaussian distribution is unconditional. We say that a random vector *X* in $R^{n}$ is unconditional if it has a probability density function $f_{X}$ with respect to Lebesgue measure in $R^{n}$ such that $f_{X}$ is unconditional.

**Theorem** **4.** *Let X be a symmetric log-concave random vector in $R^{n}$, n≥2. Then,*

(37) $$\begin{array}{c} h\left(X\right)\geq \frac{n}{2}\log\frac{|K_{X}{|}^{\frac{1}{n}}}{c(n)}, \end{array}$$

*where $|K_{X}|$ denotes the determinant of the covariance matrix of X, and $c\left(n\right)=\frac{e^{2}n^{2}}{4\sqrt{2}\left(n+2\right)}$. If, in addition, X is unconditional, then $c\left(n\right)=\frac{e^{2}}{2}$.*

By combining Theorem 4 with the well-known upper bound on the differential entropy, we deduce that, for every symmetric log-concave random vector *X* in $R^{n}$,

(38) $$\frac{n}{2}\log\left(\frac{|K_{X}{|}^{\frac{1}{n}}}{c(n)}\right)\leq h\left(X\right)\leq \frac{n}{2}\log\left(2\pi e|K_{X}{|}^{\frac{1}{n}}\right),$$

where $c\left(n\right)=\frac{e^{2}n^{2}}{4\sqrt{2}\left(n+2\right)}$ in general, and $c\left(n\right)=\frac{e^{2}}{2}$ if, in addition, *X* is unconditional.

Using Theorem 4, we immediately obtain the following upper bound for the relative entropy $D(X||G_{X})$ between a symmetric log-concave random vector *X* and a Gaussian $G_{X}$ with the same covariance matrix as that of *X*.

**Corollary** **3.** *Let X be a symmetric log-concave random vector in $R^{n}$. Then,*

(39) $$\begin{array}{c} D(X||G_{X})\leq \frac{n}{2}\log\left(2\pi ec\left(n\right)\right), \end{array}$$

*where $G_{X}∼N\left(0,K_{X}\right)$, with $c\left(n\right)=\frac{n^{2}e^{2}}{\left(n+2\right)4\sqrt{2}}$ in general, and $c\left(n\right)=\frac{e^{2}}{2}$ when X is unconditional.*

For isotropic unconditional log-concave random vectors (whose definition we recall in Section 3.3 below), we extend Theorem 4 to other moments.

**Theorem** **5.** *Let $X=(X_{1},\cdots ,X_{n})$ be an isotropic unconditional log-concave random vector. Then, for every p>−1,*

(40) $$h\left(X\right)\geq \max_{i\in \{1,\cdots ,n\}}n\log\left(\frac{2∥X_{i}{∥}_{p}}{\Gamma {\left(p+1\right)}^{\frac{1}{p}}}\frac{1}{c}\right),$$

*where $c=e\sqrt{6}$. If, in addition, $f_{X}$ is invariant under permutations of coordinates, then c=e.*

### 2.2. Extension to γ-Concave Random Variables

The bound in Theorem 1 can be extended to a larger class of random variables than log-concave, namely the class of γ-concave random variables that we describe next.

Let γ<0. We say that a probability density function $f:R^{n}\to \left[0,+\infty \right)$ is *γ-concave* if $f^{\gamma }$ is convex. Equivalently, *f* is γ-concave if for every λ∈[0,1] and every $x,y\in R^{n}$, one has

(41) $$f\left(\left(1-\lambda \right)x+\lambda y\right)\geq {\left(\left(1-\lambda \right)f{\left(x\right)}^{\gamma }+\lambda f{\left(y\right)}^{\gamma }\right)}^{\frac{1}{\gamma }}.$$

As γ→0, (41) agrees with (22), and thus 0-concave distributions corresponds to log-concave distributions. The class of γ-concave distributions has been deeply studied in [26,27].

Since for fixed a,b≥0 the function ${\left(\left(1-\lambda \right)a^{\gamma }+\lambda b^{\gamma }\right)}^{\frac{1}{\gamma }}$ is non-decreasing in γ, we deduce that any log-concave distribution is γ-concave, for any γ<0.

For example, extended Cauchy distributions, that is, distributions of the form

(42) $$f_{X}\left(x\right)=\frac{C_{\gamma }}{1+{\left|x\right|}^{n-\frac{1}{\gamma }}},\;x\in R^{n},$$

where $C_{\gamma }$ is the normalization constant, are γ-concave distributions (but are not log-concave).

We say that a random vector *X* in $R^{n}$ is γ-concave if it has a probability density function $f_{X}$ with respect to Lebesgue measure in $R^{n}$ such that $f_{X}$ is γ-concave.

We derive the following lower bound on the differential entropy for one-dimensional symmetric γ-concave random variables, with γ∈(−1,0).

**Theorem** **6.** *Let γ∈(−1,0). Let X be a symmetric γ-concave random variable. Then, for every $p\in \left(-1,-1-\frac{1}{\gamma }\right)$,*

(43) $$h\left(X\right)\geq \log\left(\frac{{2∥X∥}_{p}}{\Gamma {\left(p+1\right)}^{\frac{1}{p}}}\frac{\Gamma {\left(-1-\frac{1}{\gamma }\right)}^{1+\frac{1}{p}}}{\Gamma \left(-\frac{1}{\gamma }\right)\Gamma {\left(-\frac{1}{\gamma }-\left(p+1\right)\right)}^{\frac{1}{p}}}\right).$$

Notice that (43) reduces to (23) as γ→0. Theorem 6 implies the following relation between entropy and second moment, for any $\gamma \in (-\frac{1}{3},0)$.

**Corollary** **4.** *Let $\gamma \in (-\frac{1}{3},0)$. Let X be a symmetric γ-concave random variable. Then,*

(44) $$h\left(X\right)\geq \frac{1}{2}\log\left({2∥X∥}_{2}^{2}\frac{\Gamma {\left(-1-\frac{1}{\gamma }\right)}^{3}}{\Gamma {\left(-\frac{1}{\gamma }\right)}^{2}\Gamma \left(-\frac{1}{\gamma }-3\right)}\right)=\frac{1}{2}\log\left({2∥X∥}_{2}^{2}\frac{\left(2\gamma +1)(3\gamma +1\right)}{{\left(\gamma +1\right)}^{2}}\right).$$

### 2.3. Reverse Entropy Power Inequality with an Explicit Constant

As an application of Theorems 3 and 4, we establish in Theorems 7 and 8 below a reverse form of the entropy power inequality (7) with explicit constants, for uncorrelated log-concave random vectors. Recall the definition of the entropy power (8).

**Theorem** **7.** *Let X and Y be uncorrelated log-concave random variables. Then,*

(45) $$N\left(X+Y\right)\leq \frac{\pi e}{2}\left(N\left(X\right)+N\left(Y\right)\right).$$

As a consequence of Corollary 4, reverse entropy power inequalities for more general distributions can be obtained. In particular, for any uncorrelated symmetric γ-concave random variables *X* and *Y*, with $\gamma \in (-\frac{1}{3},0)$,

(46) $$N\left(X+Y\right)\leq \pi e\frac{{\left(\gamma +1\right)}^{2}}{\left(2\gamma +1)(3\gamma +1\right)}\left(N\left(X\right)+N\left(Y\right)\right).$$

One cannot have a reverse entropy power inequality in higher dimensions for arbitrary log-concave random vectors. Indeed, just consider *X* uniformly distributed on $\left[-\frac{\varepsilon }{2},\frac{\varepsilon }{2}\right]\times \left[-\frac{1}{2},\frac{1}{2}\right]$ and *Y* uniformly distributed on $\left[-\frac{1}{2},\frac{1}{2}\right]\times \left[-\frac{\varepsilon }{2},\frac{\varepsilon }{2}\right]$ in $R^{2}$, with ε>0 small enough so that N(X) and N(Y) are arbitrarily small compared to N(X+Y). Hence, we need to put *X* and *Y* in a certain position so that a reverse form of (7) is possible. While the isotropic position (discussed in Section 3) will work, it can be relaxed to the weaker condition that the covariance matrices are proportionals. Recall that we denote by $K_{X}$ the covariance matrix of *X*.

**Theorem** **8.** *Let X and Y be uncorrelated symmetric log-concave random vectors in $R^{n}$ such that $K_{X}$ and $K_{Y}$ are proportionals. Then,*

(47) $$N\left(X+Y\right)\leq \frac{\pi e^{3}n^{2}}{2\sqrt{2}\left(n+2\right)}\left(N\left(X\right)+N\left(Y\right)\right).$$

*If, in addition, X and Y are unconditional, then*

(48) $$N\left(X+Y\right)\leq \pi e^{3}\left(N\left(X\right)+N\left(Y\right)\right).$$

### 2.4. New Bounds on the Rate-distortion Function

As an application of Theorems 1 and 3, we show in Corollary 5 below that in the class of one-dimensional log-concave distributions, the rate-distortion function does not exceed the Shannon lower bound by more than $\log(\sqrt{\pi e})\approx 1.55$ bits (which can be refined to log(e)≈1.44 bits when the source is symmetric), independently of *d* and r≥1. Denote for brevity

(49) $$\begin{array}{cc} \beta_{r} & ≜\sqrt{1+\frac{r^{\frac{2}{r}}\Gamma \left(\frac{3}{r}\right)}{\Gamma (\frac{1}{r})}}, \end{array}$$

and recall the definition of $\alpha_{r}$ in (3).

We start by giving a bound on the difference between the rate-distortion function and the Shannon lower bound, which applies to general, not necessarily log-concave, random variables.

**Theorem** **9.** Let d≥0 and r≥1. Let X be an arbitrary random variable.

*(1) Let r∈[1,2]. If ${∥X∥}_{2}>d^{\frac{1}{r}}$, then*

(50) $$\begin{array}{c} R_{X}\;\left(d\right)-{\underline{R}}_{X}\;\left(d\right)\leq D(X||G_{X})+\log\frac{\alpha_{r}}{\sqrt{2\pi e}}. \end{array}$$

*If ${∥X∥}_{2}\leq d^{\frac{1}{r}}$, then $R_{X}\;\left(d\right)=0$.*

*(2) Let r>2. If ${∥X∥}_{2}\geq d^{\frac{1}{r}}$, then*

(51) $$\begin{array}{c} R_{X}\;\left(d\right)-{\underline{R}}_{X}\;\left(d\right)\leq D(X||G_{X})+\log\beta_{r}. \end{array}$$

*If ${∥X∥}_{r}\leq d^{\frac{1}{r}}$, then $R_{X}\;\left(d\right)=0$. If ${∥X∥}_{r}>d^{\frac{1}{r}}$ and ${∥X∥}_{2}<d^{\frac{1}{r}}$, then $R_{X}\;\left(d\right)\leq \log\frac{\sqrt{2\pi e}\beta_{r}}{\alpha_{r}}$.*

**Remark** **3.** For Gaussian X and r=2, the upper bound in *(50)* is 0, as expected.

The next result refines the bounds in Theorem 9 for symmetric log-concave random variables when r>2.

**Theorem** **10.** Let d≥0 and r>2. Let X be a symmetric log-concave random variable.

*If ${∥X∥}_{2}\geq d^{\frac{1}{r}}$, then*

(52) $$\begin{array}{c} R_{X}\;\left(d\right)-{\underline{R}}_{X}\;\left(d\right)\leq D(X||G_{X})+\min\left\{\log\left(\beta_{r}\right),\log\frac{\alpha_{r}\Gamma {\left(r+1\right)}^{\frac{1}{r}}}{2\sqrt{\pi e}}\right\}. \end{array}$$

*If ${∥X∥}_{r}\leq d^{\frac{1}{r}}$ or ${∥X∥}_{2}\leq \frac{\sqrt{2}}{\Gamma {\left(r+1\right)}^{\frac{1}{r}}}d^{\frac{1}{r}}$, then $R_{X}\;\left(d\right)=0$. If ${∥X∥}_{r}>d^{\frac{1}{r}}$ and ${∥X∥}_{2}\in \left(\frac{\sqrt{2}}{\Gamma {\left(r+1\right)}^{\frac{1}{r}}}d^{\frac{1}{r}},d^{\frac{1}{r}}\right)$, then $R_{X}\;\left(d\right)\leq \min\left\{\log\frac{\sqrt{2\pi e}\beta_{r}}{\alpha_{r}},\log\frac{\Gamma {\left(r+1\right)}^{\frac{1}{r}}}{\sqrt{2}}\right\}$.*

To bound $R_{X}\;\left(d\right)-{\underline{R}}_{X}\;\left(d\right)$ independently of the distribution of *X*, we apply the bound (35) on $D(X||G_{X})$ to Theorems 9 and 10:

**Corollary** **5.** *Let X be a log-concave random variable. For r∈[1,2], we have*

(53) $$\begin{array}{c} R_{X}\;\left(d\right)-{\underline{R}}_{X}\;\left(d\right)\leq \log\frac{\alpha_{r}}{2}. \end{array}$$

*For r>2, we have*

(54) $$\begin{array}{c} R_{X}\;\left(d\right)-{\underline{R}}_{X}\;\left(d\right)\leq \log\left(\sqrt{\frac{\pi e}{2}}\beta_{r}\right). \end{array}$$

*If, in addition, X is symmetric, then, for r>2, we have*

(55) $$\begin{array}{c} R_{X}\;\left(d\right)-{\underline{R}}_{X}\;\left(d\right)\leq \min\left\{\log\frac{\alpha_{r}\Gamma {\left(r+1\right)}^{\frac{1}{r}}}{2\sqrt{2}},\;\log\left(\sqrt{\frac{\pi e}{2}}\beta_{r}\right)\right\}. \end{array}$$

**Figure 1:**
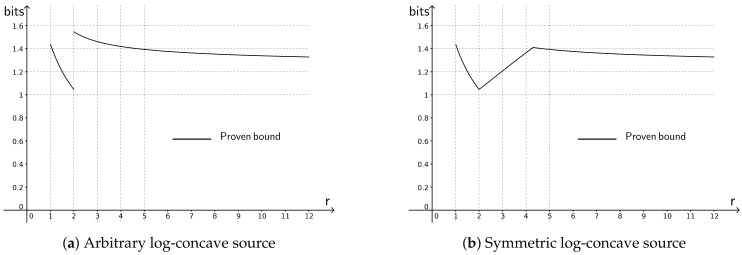
The bound on the difference between the rate-distortion function under *r*-th moment constraint and the Shannon lower bound, stated in Corollary 5.

Figure 1a presents our bound for different values of *r*. Regardless of *r* and *d*,

(56) $$R_{X}\;\left(d\right)-{\underline{R}}_{X}\;\left(d\right)\leq \log\left(\sqrt{\pi e}\right)\approx 1.55\mathrm{bits}.$$

The bounds in Figure 1a tighten for symmetric log-concave sources when r∈(2,4.3). Figure 1b presents this tighter bound for different values of *r*. Regardless of *r* and *d*,

(57) $$R_{X}\;\left(d\right)-{\underline{R}}_{X}\;\left(d\right)\leq \log(e)\approx 1.44\mathrm{bits}.$$

One can see that the graph in Figure 1b is continuous at r=2, contrary to the graph in Figure 1a. This is because Theorem 2, which applies to symmetric log-concave random variables, is strong enough to imply the tightening of (51) given in (52), while Proposition 1, which provides a counterpart of Theorem 2 applicable to all log-concave random variables, is insufficient to derive a similar tightening in that more general setting.

**Remark** **4.** *While Corollary 5 bounds the difference $R_{X}\;\left(d\right)-\underset{-}{R}{}_{X}\;\left(d\right)$ by a universal constant independent of the distribution of X, tighter bounds can be obtained if one is willing to relinquish such universality. For example, for mean-square distortion (r=2) and a uniformly distributed source U, using Remark 1, we obtain*

(58) $$\begin{array}{c} R_{U}\left(d\right)-\underset{-}{R}{}_{U}\left(d\right)\leq \frac{1}{2}\log\frac{2\pi e}{12}\approx 0.254bits. \end{array}$$

Theorem 9 easily extends to random vector *X* in $R^{n}$, n≥2, with a similar proof. The only difference being an extra term of $\frac{n}{2}\log\left(\frac{1}{n}{∥X∥}_{2}^{2}/{\left|K_{X}\right|}^{\frac{1}{n}}\right)$ that will appear on the right-hand side of (50) and (51), and will come from the upper bound on the differential entropy (38). Here,

$${∥X∥}_{p}≜{\left(E\left[\sum_{i=1}^{n}{\left|X_{i}\right|}^{p}\right]\right)}^{\frac{1}{p}}.$$

As a result, the bound $R_{X}\;\left(d\right)-\underset{-}{R}{}_{X}\;\left(d\right)$ can be arbitrarily large in higher dimensions because of the term $\frac{1}{n}{∥X∥}_{2}^{2}/{\left|K_{X}\right|}^{\frac{1}{n}}$. However, for isotropic random vectors (whose definition we recall in Section 3.3 below), one has $\frac{1}{n}{∥X∥}_{2}^{2}={\left|K_{X}\right|}^{\frac{1}{n}}$. Hence, using the bound (39) on $D(X||G_{X})$, we can bound $R_{X}\;\left(d\right)-{\underline{R}}_{X}\;\left(d\right)$ independently of the distribution of isotropic log-concave random vector *X* in $R^{n}$, n≥2.

**Corollary** **6.** *Let X be an isotropic log-concave random vector in $R^{n}$, n≥2. Then,*

(59) $$R_{X}\;\left(d\right)-{\underline{R}}_{X}\;\left(d\right)\leq \frac{n}{2}\log\left(2\pi e\;c\left(n\right)\right),$$

*where $c\left(n\right)=\frac{n^{2}e^{2}}{\left(n+2\right)4\sqrt{2}}$ in general, and $c\left(n\right)=\frac{e^{2}}{2}$ if, in addition, X is unconditional.*

Let us consider the rate-distortion function under the determinant constraint for random vectors in $R^{n}$, n≥2:

(60) $$R_{X}^{cov}\left(d\right)=\underset{P_{\hat{X}|X}:{\left|K_{X-\hat{X}}\right|}^{\frac{1}{n}}\leq d}{\inf}I\left(X;\hat{X}\right),$$

where the infimum is taken over all joint distributions satisfying the determinant constraint $|K_{X-\hat{X}}{|}^{\frac{1}{n}}\leq \;d$. For this distortion measure, we have the following bound.

**Theorem** **11.** *Let X be a symmetric log-concave random vector in $R^{n}$. If $|K_{X}{|}^{\frac{1}{n}}>d$, then*

(61) $$0\leq R_{X}^{cov}\left(d\right)-{\underline{R}}_{X}\;\left(d\right)\leq D(X||G_{X})\leq \frac{n}{2}\log\left(2\pi e\;c\left(n\right)\right),$$

*with $c\left(n\right)=\frac{n^{2}e^{2}}{\left(n+2\right)4\sqrt{2}}$. If, in addition, X is unconditional, then $c\left(n\right)=\frac{e^{2}}{2}$. If $|K_{X}{|}^{\frac{1}{n}}\leq d$, then $R_{X}^{cov}\left(d\right)=0$.*

### 2.5. New Bounds on the Capacity of Memoryless Additive Channels

As another application of Theorem 3, we compare the capacity $C_{Z}$ of a channel with log-concave additive noise *Z* with the capacity of the Gaussian channel. Recall that the capacity of the Gaussian channel is

(62) $$\underset{-}{C}{}_{Z}\left(P\right)=\frac{1}{2}\log\left(1+\frac{P}{\mathrm{Var}[Z]}\right).$$

**Theorem** **12.** *Let Z be a log-concave random variable. Then,*

(63) $$0\leq C_{Z}\left(P\right)-\underset{-}{C}{}_{Z}\left(P\right)\leq \log\sqrt{\frac{\pi e}{2}}\approx 1.05\;\;\mathrm{bits}.$$

**Remark** **5.** Theorem 12 tells us that the capacity of a channel with log-concave additive noise exceeds the capacity a Gaussian channel by no more than 1.05 bits.

As an application of Theorem 4, we can provide bounds for the capacity of a channel with log-concave additive noise *Z* in $R^{n}$, n≥1. The formula for capacity (18) generalizes to dimension *n* as

(64) $$C_{Z}\left(P\right)=\underset{X:\frac{1}{n}{∥X∥}_{2}^{2}\leq P}{\sup}I\left(X;X+Z\right).$$

**Theorem** **13.** *Let Z be a symmetric log-concave random vector in $R^{n}$. Then,*

(65) $$0\leq C_{Z}\left(P\right)-\frac{n}{2}\log\left(1+\frac{P}{|K_{Z}{|}^{\frac{1}{n}}}\right)\leq \frac{n}{2}\log\left(2\pi e\;c\left(n\right)\left(\frac{\frac{1}{n}{∥Z∥}_{2}^{2}+P}{|K_{Z}{|}^{\frac{1}{n}}+P}\right)\right),$$

*where $c\left(n\right)=\frac{n^{2}e^{2}}{\left(n+2\right)4\sqrt{2}}$. If, in addition, Z is unconditional, then $c\left(n\right)=\frac{e^{2}}{2}$.*

The upper bound in Theorem 13 can be arbitrarily large by inflating the ratio $\frac{1}{n}{∥X∥}_{2}^{2}/{\left|K_{X}\right|}^{\frac{1}{n}}$. For isotropic random vectors (whose definition is recalled in Section 3.3 below), one has $\frac{1}{n}{∥Z∥}_{2}^{2}={\left|K_{Z}\right|}^{\frac{1}{n}}$, and the following corollary follows.

**Corollary** **7.** *Let Z be an isotropic log-concave random vector in $R^{n}$. Then,*

(66) $$0\leq C_{Z}\left(P\right)-\frac{n}{2}\log\left(1+\frac{P}{|K_{Z}{|}^{\frac{1}{n}}}\right)\leq \frac{n}{2}\log\left(2\pi e\;c(n)\right),$$

*where $c\left(n\right)=\frac{n^{2}e^{2}}{\left(n+2\right)4\sqrt{2}}$. If, in addition, Z is unconditional, then $c\left(n\right)=\frac{e^{2}}{2}$.*

## 3. New Lower Bounds on the Differential Entropy

### 3.1. Proof of Theorem 1

The key to our development is the following result for one-dimensional log-concave distributions, well-known in convex geometry. It can be found in [28], in a slightly different form.

**Lemma** **1.** *The function*

(67) $$\begin{array}{c} F\left(r\right)=\frac{1}{\Gamma (r+1)}\int_{0}^{+\infty }x^{r}f\left(x\right)\;dx \end{array}$$

*is log-concave on [−1,+∞), whenever f:[0;+∞)→[0;+∞) is log-concave [28].*

**Proof** **of** **Theorem** **1.** Let p>0. Applying Lemma 1 to the values −1,0,p, we have

(68) $$\begin{array}{c} F\left(0\right)=F\left(\frac{p}{p+1}\left(-1\right)+\frac{1}{p+1}p\right)\geq F{\left(-1\right)}^{\frac{p}{p+1}}F{\left(p\right)}^{\frac{1}{p+1}}. \end{array}$$

The bound in Theorem 1 follows by computing the values F(−1), F(0) and F(p) for $f=f_{X}$.

One has

(69) $$\begin{array}{c} F\left(0\right)=\frac{1}{2},\;F\left(p\right)=\frac{{∥X∥}_{p}^{p}}{2\Gamma (p+1)}. \end{array}$$

To compute F(−1), we first provide a different expression for F(r). Notice that

(70) $$F\left(r\right)=\frac{1}{\Gamma (r+1)}\int_{0}^{+\infty }x^{r}\int_{0}^{f_{X}\left(x\right)}\;dt\;dx=\frac{r+1}{\Gamma (r+2)}\int_{0}^{\max f_{X}}\int_{\left\{x\geq 0:f_{X}\left(x\right)\geq t\right\}}x^{r}\;dx\;dt.$$

Denote the generalized inverse of $f_{X}$ by $f_{X}^{-1}\left(t\right)≜\sup\left\{x\geq 0:f_{X}\left(x\right)\geq t\right\}$, t≥0. Since $f_{X}$ is log-concave and

(71) $$f_{X}\left(x\right)\leq f_{X}\left(0\right)=\max f_{X},$$

it follows that $f_{X}$ is non-increasing on [0,+∞). Therefore, $\left\{x\geq 0:f_{X}\left(x\right)\geq t\right\}=\left[0,f_{X}^{-1}\left(t\right)\right]$. Hence,

(72) $$\begin{array}{c} F\left(r\right)=\frac{r+1}{\Gamma (r+2)}\int_{0}^{f_{X}\left(0\right)}\int_{0}^{f_{X}^{-1}\left(t\right)}x^{r}\;dx\;dt=\frac{1}{\Gamma (r+2)}\int_{0}^{f_{X}\left(0\right)}{\left(f_{X}^{-1}\left(t\right)\right)}^{r+1}\;dt. \end{array}$$

We deduce that

(73) $$\begin{array}{c} F\left(-1\right)=f_{X}\left(0\right). \end{array}$$

Plugging (69) and (73) into (68), we obtain

(74) $$\begin{array}{c} f_{X}\left(0\right)\leq \frac{\Gamma {\left(p+1\right)}^{\frac{1}{p}}}{{2∥X∥}_{p}}. \end{array}$$

It follows immediately that

(75) $$\begin{array}{c} h\left(X\right)=\int f_{X}\left(x\right)\log\frac{1}{f_{X}\left(x\right)}\;dx\geq \log\frac{1}{f_{X}\left(0\right)}\geq \log\frac{{2∥X∥}_{p}}{\Gamma {\left(p+1\right)}^{\frac{1}{p}}}. \end{array}$$

For p∈(−1,0), the bound is obtained similarly by applying Lemma 1 to the values −1,p,0.

We now show that equality is attained, by letting p↓−1, by *U* uniformly distributed on a symmetric interval $\left[-\frac{a}{2},\frac{a}{2}\right]$, for some a>0. In this case, we have

(76) $${∥U∥}_{p}^{p}={\left(\frac{a}{2}\right)}^{p}\frac{1}{p+1}.$$

Hence,

(77) $$\frac{1}{p}\log\frac{2^{p}{∥U∥}_{p}^{p}}{\Gamma (p+1)}=\log\frac{a}{\Gamma {\left(p+2\right)}^{\frac{1}{p}}}\underset{p\to -1}{\to }\log\left(a\right)=h\left(U\right).$$

☐

**Remark** **6.** *From *(71)* and *(74)*, we see that the following statement holds: For every symmetric log-concave random variable $X∼f_{X}$, for every p>−1, and for every x∈R,*

(78) $$\begin{array}{c} f_{X}\left(x\right)\leq \frac{\Gamma {\left(p+1\right)}^{\frac{1}{p}}}{{2∥X∥}_{p}}. \end{array}$$

*Inequality *(78)* is the main ingredient in the proof of Theorem 1. It is instructive to provide a direct proof of inequality *(78)* without appealing to Lemma 1, the ideas going back to [25]:*

**Proof** **of** **inequality** **(78)** By considering X|X≥0, where X is symmetric log-concave, it is enough to show that for every log-concave density f supported on [0,+∞), one has

(79) $$f\left(0\right){\left(\int_{0}^{+\infty }x^{p}f\left(x\right)\;dx\right)}^{\frac{1}{p}}\leq \Gamma {\left(p+1\right)}^{\frac{1}{p}}.$$

By a scaling argument, one may assume that f(0)=1. Take $g\left(x\right)=e^{-x}$. If f=g, then the result follows by a straightforward computation. Assume that f≠g. Since f≠g and ∫f=∫g, the function f−g changes sign at least one time. However, since f(0)=g(0), f is log-concave and g is log-affine, the function f−g changes sign exactly once. It follows that there exists a unique point $x_{0}>0$ such that for every $0<x<x_{0}$, f(x)≥g(x), and for every $x>x_{0}$, f(x)≤g(x). We deduce that for every x>0, and p≠0,

(80) $$\frac{1}{p}\left(f\left(x\right)-g\left(x\right)\right)\left(x^{p}-x_{0}^{p}\right)\leq 0.$$

*Integrating over x>0, we arrive at*

(81) $$\frac{1}{p}\left(\int_{0}^{+\infty }x^{p}f\left(x\right)\;dx-\Gamma \left(p+1\right)\right)=\frac{1}{p}\int_{0}^{+\infty }\left(x^{p}-x_{0}^{p}\right)\left(f\left(x\right)-g\left(x\right)\right)\;dx\leq 0,$$

*which yields the desired result.* ☐

*Actually, the powerful and versatile result of Lemma 1, which implies *(78)*, is also proved using the technique in *(79)–(81)*. In the context of information theory, Lemma 1 has been previously applied to obtain reverse entropy power inequalities [7], as well as to establish optimal concentration of the information content [29]. In this paper, we make use of Lemma 1 to prove Theorem 1. Moreover, Lemma 1 immediately implies Theorem 2. Below, we recall the argument for completeness.*

**Proof** **of** **Theorem** **2.** The result follows by applying Lemma 1 to the values 0,p,q. If 0<p<q, then

(82) $$F\left(p\right)=F\left(0\cdot \left(1-\frac{p}{q}\right)+q\cdot \left(\frac{p}{q}\right)\right)\geq F{\left(0\right)}^{1-\frac{p}{q}}F{\left(q\right)}^{\frac{p}{q}}.$$

Hence,

(83) $$\frac{{∥X∥}_{p}^{p}}{\Gamma (p+1)}\geq {\left(\frac{{∥X∥}_{q}^{q}}{\Gamma (q+1)}\right)}^{\frac{p}{q}},$$

which yields the desired result. The bound is obtained similarly if p<q<0 or if p<0<q. ☐

### 3.2. Proof of Theorem 3 and Proposition 1

The proof leverages the ideas from [10].

**Proof** **of Theorem 3.** Let *Y* be an independent copy of *X*. Jensen’s inequality yields

(84) $$h\left(X\right)=-\int f_{X}\log\left(f_{X}\right)\geq -\log\left(\int f_{X}^{2}\right)=-\log\left(f_{X-Y}\left(0\right)\right).$$

Since X−Y is symmetric and log-concave, we can apply inequality (74) to X−Y to obtain

(85) $$\frac{1}{f_{X-Y}\left(0\right)}\geq \frac{{2∥X-Y∥}_{p}}{\Gamma {\left(p+1\right)}^{\frac{1}{p}}}\geq \frac{{2∥X-E\left[X\right]∥}_{p}}{\Gamma {\left(p+1\right)}^{\frac{1}{p}}},$$

where the last inequality again follows from Jensen’s inequality. Combining (84) and (85) leads to the desired result:

(86) $$h\left(X\right)\geq \log\left(\frac{1}{f_{X-Y}\left(0\right)}\right)\geq \log\left(\frac{{2∥X-E\left[X\right]∥}_{p}}{\Gamma {\left(p+1\right)}^{\frac{1}{p}}}\right).$$

For p=2, one may tighten (85) by noticing that

(87) $${∥X-Y∥}_{2}^{2}=2\mathrm{Var}\left[X\right].$$

Hence,

(88) $$h\left(X\right)\geq \log\left(\frac{1}{f_{X-Y}\left(0\right)}\right)\geq \log\left(\sqrt{2}{∥X-Y∥}_{2}\right)=\log\left(2\sqrt{\mathrm{Var}[X]}\right).$$

☐

**Proof** **of** **Proposition** **1.** Let *Y* be an independent copy of *X*. Since X−Y is symmetric and log-concave, we can apply Theorem 2 to X−Y. Jensen’s inequality and triangle inequality yield:

(89) $${∥X-E\left[X\right]∥}_{q}\leq {∥X-Y∥}_{q}\leq \frac{\Gamma {\left(q+1\right)}^{\frac{1}{q}}}{\Gamma {\left(p+1\right)}^{\frac{1}{p}}}{∥X-Y∥}_{p}\leq 2\frac{\Gamma {\left(q+1\right)}^{\frac{1}{q}}}{\Gamma {\left(p+1\right)}^{\frac{1}{p}}}{∥X-E\left[X\right]∥}_{p}.$$

☐

### 3.3. Proof of Theorem 4

We say that a random vector $X∼f_{X}$ is *isotropic* if *X* is symmetric and for all unit vectors θ, one has

(90) $$\begin{array}{c} E\left[{\langle X,\theta \rangle }^{2}\right]=m_{X}^{2}, \end{array}$$

for some constant $m_{X}>0$. Equivalently, *X* is isotropic if its covariance matrix $K_{X}$ is a multiple of the identity matrix $I_{n}$,

(91) $$K_{X}=m_{X}^{2}I_{n},$$

for some constant $m_{X}>0$. The constant

(92) $$\begin{array}{c} \ell_{X}≜f_{X}{\left(0\right)}^{\frac{1}{n}}m_{X} \end{array}$$

is called the *isotropic constant* of *X*.

It is well known that $\ell_{X}$ is bounded from below by a positive constant independent of the dimension [30]. A long-standing conjecture in convex geometry, the *hyperplane conjecture*, asks whether the isotropic constant of an isotropic log-concave random vector is also bounded from above by a universal constant (independent of the dimension). This conjecture holds under additional assumptions, but, in full generality, $\ell_{X}$ is known to be bounded only by a constant that depends on the dimension. For further details, we refer the reader to [31]. We will use the following upper bounds on $\ell_{X}$ (see [32] for the best dependence on the dimension up to date).

**Lemma** **2.** Let X be an isotropic log-concave random vector in $R^{n}$, with n≥2. Then, $\ell_{X}^{2}\leq \frac{n^{2}e^{2}}{\left(n+2\right)4\sqrt{2}}$. If, in addition, X is unconditional, then $\ell_{X}^{2}\leq \frac{e^{2}}{2}$.

*If X is uniformly distributed on a convex set, these bounds hold without factor* $e^{2}$.

Even though the bounds in Lemma 2 are well known, we could not find a reference in the literature. We thus include a short proof for completeness.

**Proof.**  It was shown by Ball [30] (Lemma 8) that if *X* is uniformly distributed on a convex set, then $\ell_{X}^{2}\leq \frac{n^{2}}{\left(n+2\right)4\sqrt{2}}$. If *X* is uniformly distributed on a convex set and is unconditional, then it is known that $\ell_{X}^{2}\leq \frac{1}{2}$ (see e.g., [33] (Proposition 2.1)). Now, one can pass from uniform distributions on a convex set to log-concave distributions at the expense of an extra factor $e^{2}$, as shown by Ball [30] (Theorem 7). ☐

We are now ready to prove Theorem 4.

**Proof** **of** **Theorem** **4.** Let $\tilde{X}∼f_{\tilde{X}}$ be an isotropic log-concave random vector. Notice that $f_{\tilde{X}}{\left(0\right)}^{\frac{2}{n}}{\left|K_{\tilde{X}}\right|}^{\frac{1}{n}}=\ell_{\tilde{X}}^{2}$, hence, using Lemma 2, we have

(93) $$\begin{array}{c} h\left(\tilde{X}\right)=\int f_{\tilde{X}}\left(x\right)\log\frac{1}{f_{\tilde{X}}\left(x\right)}dx\geq \log\frac{1}{f_{\tilde{X}}\left(0\right)}\geq \frac{n}{2}\log\frac{|K_{\tilde{X}}{|}^{\frac{1}{n}}}{c(n)}, \end{array}$$

with $c\left(n\right)=\frac{n^{2}e^{2}}{\left(n+2\right)4\sqrt{2}}$. If, in addition, $\tilde{X}$ is unconditional, then again by Lemma 2, $c\left(n\right)=\frac{e^{2}}{2}$.

Now consider an arbitrary symmetric log-concave random vector *X*. One can apply a change of variable to put *X* in isotropic position. Indeed, by defining $\tilde{X}=K_{X}^{-\frac{1}{2}}X$, one has for every unit vector θ,

(94) $$\begin{array}{c} E\left[{\langle \tilde{X},\theta \rangle }^{2}\right]=E\left[{\langle X,K_{X}^{-\frac{1}{2}}\theta \rangle }^{2}\right]=\langle K_{X}\left(K_{X}^{-\frac{1}{2}}\theta \right),K_{X}^{-\frac{1}{2}}\theta \rangle =1. \end{array}$$

It follows that $\tilde{X}$ is an isotropic log-concave random vector with isotropic constant 1. Therefore, we can use (93) to obtain

(95) $$\begin{array}{c} h\left(\tilde{X}\right)\geq \frac{n}{2}\log\frac{1}{c(n)}, \end{array}$$

where $c\left(n\right)=\frac{n^{2}e^{2}}{\left(n+2\right)4\sqrt{2}}$ in general, and $c\left(n\right)=\frac{e^{2}}{2}$ when *X* is unconditional. We deduce that

(96) $$\begin{array}{c} h\left(X\right)=h\left(\tilde{X}\right)+\frac{n}{2}\log{\left|K_{X}\right|}^{\frac{1}{n}}\geq \frac{n}{2}\log\frac{|K_{X}{|}^{\frac{1}{n}}}{c(n)}. \end{array}$$

☐

### 3.4. Proof of Theorem 5

First, we need the following lemma.

**Lemma** **3.** *Let $X∼f_{X}$ be an isotropic unconditional log-concave random vector. Then, for every i∈{1,⋯,n},*

(97) $$f_{X_{i}}\left(0\right)\geq \frac{f_{X}{\left(0\right)}^{\frac{1}{n}}}{c},$$

*where $f_{X_{i}}$ is the marginal distribution of the i-th component of X, i.e., for every t∈R,*

(98) $$f_{X_{i}}\left(t\right)=\int_{R^{n-1}}f_{X}\left(x_{1},\cdots ,x_{i-1},t,x_{i+1},\cdots ,x_{n}\right)dx_{1}\cdots dx_{i-1}dx_{i+1}\cdots dx_{n}.$$

*Here, $c=e\sqrt{6}$. If, in addition, $f_{X}$ is invariant under permutations of coordinates, then c=e [33] (Proposition 3.2).*

**Proof** **of** **Theorem** **5.** Let i∈{1,⋯,n}. We have

(99) $$∥X_{i}{∥}_{p}^{p}=\int_{R}{\left|t\right|}^{p}f_{X_{i}}\left(t\right)\;dt.$$

Since $f_{X}$ is unconditional and log-concave, it follows that $f_{X_{i}}$ is symmetric and log-concave, so inequality (74) applies to $f_{X_{i}}$:

(100) $$\int_{R}{\left|t\right|}^{p}f_{X_{i}}\left(t\right)\;dt\leq \frac{\Gamma (p+1)}{2^{p}f_{X_{i}}{\left(0\right)}^{p}}.$$

We apply Lemma 3 to pass from $f_{X_{i}}$ to $f_{X}$ in the right side of (100):

(101) $$f_{X}{\left(0\right)}^{\frac{1}{n}}{∥X_{i}∥}_{p}\leq \frac{\Gamma {\left(p+1\right)}^{\frac{1}{p}}c}{2}.$$

Thus,

(102) $$h\left(X\right)\geq \log\frac{1}{f_{X}\left(0\right)}\geq n\log\frac{2∥X_{i}{∥}_{p}}{\Gamma {\left(p+1\right)}^{\frac{1}{p}}c}.$$

☐

## 4. Extension to γ-Concave Random Variables

In this section, we prove Theorem 6, which extends Theorem 1 to the class of γ-concave random variables, with γ<0. First, we need the following key lemma, which extends Lemma 1.

**Lemma** **4.** *Let f:[0,+∞)→[0,+∞) be a γ-concave function, with γ<0. Then, the function*

(103) $$F\left(r\right)=\frac{\Gamma (-\frac{1}{\gamma })}{\Gamma (-\frac{1}{\gamma }-\left(r+1\right))}\frac{1}{\Gamma (r+1)}\int_{0}^{+\infty }t^{r}f\left(t\right)\;dt$$

*is log-concave on $\left[-1,-1-\frac{1}{\gamma }\right)$ [34] (Theorem 7).*

One can recover Lemma 1 from Lemma 4 by letting γ tend to 0 from below.

**Proof** **of** **Theorem** **6.** Let us first consider the case p∈(−1,0). Let us denote by $f_{X}$ the probability density function of *X*. By applying Lemma 4 to the values −1,p,0, we have

$$F\left(p\right)=F\left(-1\cdot \left(-p\right)+0\cdot \left(p+1\right)\right)\geq F{\left(-1\right)}^{-p}F{\left(0\right)}^{p+1}.$$

From the proof of Theorem 1, we deduce that $F\left(-1\right)=f_{X}\left(0\right)$. In addition, notice that, for γ∈(−1,0),

(104) $$F\left(0\right)=\frac{1}{2}\frac{\Gamma (-\frac{1}{\gamma })}{\Gamma (-\frac{1}{\gamma }-1)}.$$

Hence,

(105) $$f_{X}{\left(0\right)}^{-p}\leq \frac{2^{p}{∥X∥}_{p}^{p}}{\Gamma (p+1)}\frac{\Gamma {\left(-1-\frac{1}{\gamma }\right)}^{p+1}}{\Gamma {\left(-\frac{1}{\gamma }\right)}^{p}\Gamma \left(-\frac{1}{\gamma }-\left(p+1\right)\right)},$$

and the bound on differential entropy follows:

(106) $$h\left(X\right)\geq \log\frac{1}{f_{X}\left(0\right)}\geq \frac{1}{p}\log\left(\frac{2^{p}{∥X∥}_{p}^{p}}{\Gamma (p+1)}\frac{\Gamma {\left(-1-\frac{1}{\gamma }\right)}^{p+1}}{\Gamma {\left(-\frac{1}{\gamma }\right)}^{p}\Gamma \left(-\frac{1}{\gamma }-\left(p+1\right)\right)}\right).$$

For the case $p\in \left(0,-1-\frac{1}{\gamma }\right)$, the bound is obtained similarly by applying Lemma 4 to the values −1,0,p. ☐

## 5. Reverse Entropy Power Inequality with Explicit Constant

### 5.1. Proof of Theorem 7

**Proof.**  Using the upper bound on the differential entropy (1), we have

(107) $$h\left(X+Y\right)\leq \frac{1}{2}\log\left(2\pi e\mathrm{Var}\left[X+Y\right]\right)=\frac{1}{2}\log\left(2\pi e\left(\mathrm{Var}\left[X\right]+\mathrm{Var}\left[Y\right]\right)\right),$$

the last equality being valid since *X* and *Y* are uncorrelated. Hence,

(108) N(X+Y)≤2πe(Var[X]+Var[Y]).

Using inequality (32), we conclude that

(109) $$N\left(X+Y\right)\leq \frac{\pi e}{2}\left(N\left(X\right)+N\left(Y\right)\right).$$

☐

### 5.2. Proof of Theorem 8

**Proof.**  Since *X* and *Y* are uncorrelated and $K_{X}$ and $K_{Y}$ are proportionals,

(110) $$|K_{X+Y}{|}^{\frac{1}{n}}=|K_{X}+K_{Y}{|}^{\frac{1}{n}}=|K_{X}{|}^{\frac{1}{n}}+{\left|K_{Y}\right|}^{\frac{1}{n}}.$$

Using (110) and the upper bound on the differential entropy (38), we obtain

(111) $$h\left(X+Y\right)\leq \frac{n}{2}\log\left(2\pi e|K_{X+Y}{|}^{\frac{1}{n}}\right)=\frac{n}{2}\log\left(2\pi e\left(|K_{X}{|}^{\frac{1}{n}}+{\left|K_{Y}\right|}^{\frac{1}{n}}\right)\right).$$

Using Theorem 4, we conclude that

(112) $$N\left(X+Y\right)\leq 2\pi e\left(|K_{X}{|}^{\frac{1}{n}}+{\left|K_{Y}\right|}^{\frac{1}{n}}\right)\leq 2\pi e\;c\left(n\right)\left(N\left(X\right)+N\left(Y\right)\right),$$

where $c\left(n\right)=\frac{e^{2}n^{2}}{4\sqrt{2}\left(n+2\right)}$ in general, and $c\left(n\right)=\frac{e^{2}}{2}$ if *X* and *Y* are unconditional. ☐

## 6. New Bounds on the Rate-Distortion Function

### 6.1. Proof of Theorem 9

**Proof.**  Under mean-square error distortion (r=2), the result is implicit in [21] (Chapter 10). Denote for brevity $\sigma ={∥X∥}_{2}$.

(1) Let r∈[1,2]. Assume that $\sigma >d^{\frac{1}{r}}$. We take

(113) $$\hat{X}=\left(1-d^{\frac{2}{r}}/\sigma^{2}\right)\left(X+Z\right),$$

where $Z∼N\left(0,\frac{\sigma^{2}d^{\frac{2}{r}}}{\sigma^{2}-d^{\frac{2}{r}}}\right)$ is independent of *X*. This choice of $\hat{X}$ is admissible since

(114) $$\begin{array}{c} ∥X-\hat{X}{∥}_{r}^{r}\leq {∥X-\hat{X}∥}_{2}^{r}={\left[{\left(\frac{d^{\frac{2}{r}}}{\sigma^{2}}\right)}^{2}\sigma^{2}+{\left(1-\frac{d^{\frac{2}{r}}}{\sigma^{2}}\right)}^{2}{∥Z∥}_{2}^{2}\right]}^{\frac{r}{2}}=d, \end{array}$$

where we used r≤2 and the left-hand side of inequality (26). Upper-bounding the rate-distortion function by the mutual information between *X* and $\hat{X}$, we obtain

(115) $$\begin{array}{c} R_{X}\;\left(d\right)\leq I\left(X;\hat{X}\right)=h\left(X+Z\right)-h\left(Z\right), \end{array}$$

where we used homogeneity of differential entropy for the last equality. Invoking the upper bound on the differential entropy (1), we have

(116) $$h\left(X+Z\right)-h\left(Z\right)\leq \frac{1}{2}\log\left(2\pi e\left(\sigma^{2}+\frac{\sigma^{2}d^{\frac{2}{r}}}{\sigma^{2}-d^{\frac{2}{r}}}\right)\right)-h\left(Z\right)={\underline{R}}_{X}\;\left(d\right)+D(X||G_{X})+\log\frac{\alpha_{r}}{\sqrt{2\pi e}},$$

and (50) follows.

If ${∥X∥}_{2}\leq d^{\frac{1}{r}}$, then ${∥X∥}_{r}\leq {∥X∥}_{2}\leq d^{\frac{1}{r}}$, and setting $\hat{X}\equiv 0$ leads to $R_{X}\;\left(d\right)=0$.

(2) Let r>2. The argument presented here works for every r≥1. However, for r∈[1,2], the argument in part (1) provides a tighter bound. Assume that $\sigma \geq d^{\frac{1}{r}}$. We take

(117) $$\hat{X}=X+Z,$$

where *Z* is independent of *X* and realizes the maximum differential entropy under the *r*-th moment constraint, ${∥Z∥}_{r}^{r}=d$. The probability density function of *Z* is given by

(118) $$f_{Z}\left(x\right)=\frac{r^{1-\frac{1}{r}}}{2\Gamma \left(\frac{1}{r}\right)d^{\frac{1}{r}}}e^{-\frac{{\left|x\right|}^{r}}{rd}},\;x\in R.$$

Notice that

(119) $${∥Z∥}_{2}^{2}=d^{\frac{2}{r}}\frac{r^{\frac{2}{r}}\Gamma \left(\frac{3}{r}\right)}{\Gamma (\frac{1}{r})}.$$

We have

(120) $$\begin{array}{cc} h(X+Z)-h(Z)\leq & \;\frac{1}{2}\log(2\pi e(\sigma^{2}+{∥Z∥}_{2}^{2}))-\log\left(\alpha_{r}d^{\frac{1}{r}}\right) \end{array}$$

(121) $$\begin{array}{cc} \leq & \;{\underline{R}}_{X}\;\left(d\right)+\log\left(\sqrt{2\pi e}\beta_{r}\sigma \right)-h\left(X\right), \end{array}$$

where $\beta_{r}$ is defined in (49). Hence,

(122) $$R_{X}\;\left(d\right)-{\underline{R}}_{X}\;\left(d\right)\leq D(X||G_{X})+\log\beta_{r}.$$

If ${∥X∥}_{r}^{r}\leq d$, then setting $\hat{X}\equiv 0$ leads to $R_{X}\;\left(d\right)=0$. Finally, if ${∥X∥}_{r}^{r}>d$ and $\sigma <d^{\frac{1}{r}}$, then, from (120), we obtain

(123) $$R_{X}\;\left(d\right)\leq \log\left(\sqrt{2\pi e}\beta_{r}d^{\frac{1}{r}}\right)-\log\left(\alpha_{r}d^{\frac{1}{r}}\right)=\log\frac{\sqrt{2\pi e}\beta_{r}}{\alpha_{r}}.$$

☐

### 6.2. Proof of Theorem 10

**Proof.**  Denote for brevity $\sigma ={∥X∥}_{2}$, and recall that *X* is a symmetric log-concave random variable.

Assume that $\sigma \geq d^{\frac{1}{r}}$. We take

(124) $$\begin{array}{c} \hat{X}=\left(1-\frac{\delta }{\sigma^{2}}\right)\left(X+Z\right),\;\delta ≜\frac{2}{\Gamma {\left(r+1\right)}^{\frac{2}{r}}}d^{\frac{2}{r}}, \end{array}$$

where $Z∼N\left(0,\frac{\sigma^{2}\delta }{\sigma^{2}-\delta }\right)$ is independent of *X*. This choice of $\hat{X}$ is admissible since

(125) $$\begin{array}{c} ∥X-\hat{X}{∥}_{r}^{r}\leq {∥X-\hat{X}∥}_{2}^{r}\frac{\Gamma (r+1)}{2^{\frac{r}{2}}}=\delta^{\frac{r}{2}}\frac{\Gamma (r+1)}{2^{\frac{r}{2}}}=d, \end{array}$$

where we used r>2 and Theorem 2. Using the upper bound on the differential entropy (1), we have

(126) $$\begin{array}{c} h\left(X+Z\right)-h\left(Z\right)\leq \frac{1}{2}\log\left(2\pi e\left(\sigma^{2}+\frac{\sigma^{2}\delta }{\sigma^{2}-\delta }\right)\right)-h\left(Z\right)=\frac{1}{2}\log\frac{\sigma^{2}}{\delta }. \end{array}$$

Hence,

(127) $$R_{X}\;\left(d\right)-\underset{-}{R}{}_{X}\;\left(d\right)\leq D(X||G_{X})+\log\frac{\alpha_{r}\Gamma {\left(r+1\right)}^{\frac{1}{r}}}{2\sqrt{\pi e}}.$$

If $\sigma^{2}\leq \delta$, then from Theorem 2 ${∥X∥}_{r}^{r}\leq d$, hence $R_{X}\;\left(d\right)=0$. Finally, if ${∥X∥}_{r}^{r}>d$ and $\sigma^{2}\in \left(\delta ,d^{\frac{2}{r}}\right)$, then, from (126), we obtain

(128) $$\begin{array}{c} R_{X}\;\left(d\right)\leq \frac{1}{2}\log\frac{\sigma^{2}}{\delta }\leq \frac{1}{2}\log\frac{\Gamma {\left(r+1\right)}^{\frac{2}{r}}}{2}. \end{array}$$

☐

**Remark** **7.** 1) Let us explain the strategy in the proof of Theorems 9 and 10. By definition, $R_{X}\;\left(d\right)\leq I\left(X;\hat{X}\right)$ for any $\hat{X}$ satisfying the constraint. In our study, we chose $\hat{X}$ of the form λ(X+Z), with λ∈[0,1], where Z is independent of X. To find the best bounds possible with this choice of $\hat{X}$, we need to minimize $∥X-\hat{X}{∥}_{r}^{r}$ over λ. Notice that if $\hat{X}=\lambda \left(X+Z\right)$ and Z symmetric, then $∥X-\hat{X}{∥}_{r}^{r}={∥\left(1-\lambda \right)X+\lambda Z∥}_{r}^{r}$.

*To estimate ${∥\left(1-\lambda \right)X+\lambda Z∥}_{r}^{r}$ in terms of ${∥X∥}_{r}$ and ${∥Z∥}_{r}$, one can use triangle inequality and the convexity of ${∥\cdot ∥}^{r}$ to get the bound*

(129) $${∥\left(1-\lambda \right)X+\lambda Z∥}_{r}^{r}\leq 2^{r-1}{(\left(1-\lambda \right)}^{r}{∥X∥}_{r}^{r}+\lambda^{r}{∥Z∥}_{r}^{r}),$$

*or one can apply Jensen’s inequality directly to get the bound*

(130) $${∥\left(1-\lambda \right)X+\lambda Z∥}_{r}^{r}\leq {\left(1-\lambda \right)∥X∥}_{r}^{r}+\lambda {∥Z∥}_{r}^{r}.$$

*A simple study shows that *(130)* provides a tighter bound over *(129)*. This justifies choosing $\hat{X}$ as in *(117)* in the proof of *(51)*.*

*To justify the choice of $\hat{X}$ in *(113)* (also in *(124)*), which leads the tightening of *(51)* for r∈[1,2] in *(50)* (also in *(52)*), we bound r-th norm by second norm, and we note that by the independence of X and Z,*

(131) $${∥\left(1-\lambda \right)X+\lambda Z∥}_{2}^{2}\leq {\left(1-\lambda \right)}^{2}{∥X∥}_{2}^{2}+\lambda^{2}{∥Z∥}_{2}^{2}.$$

*A simple study shows that *(131)* provides a tighter bound over *(130)*.*

*2) Using Corollary 2, if r=2, one may rewrite our bound in terms of the rate-distortion function of a Gaussian source as follows:*

(132) $$\begin{array}{c} R_{X}\;\left(d\right)\geq R_{G_{X}}\left(d\right)-\log\sqrt{\pi e}-\Delta_{p}, \end{array}$$

*where $\Delta_{p}$ is defined in *(28)*, and where*

(133) $$R_{G_{X}}\left(d\right)=\frac{1}{2}\log\frac{\sigma^{2}}{d}$$

*is the rate-distortion function of a Gaussian source with the same variance $\sigma^{2}$ as X. It is well known that for arbitrary source and mean-square distortion (see e.g., [21] (Chapter 10))*

(134) $$R_{X}\;\left(d\right)\leq R_{G_{X}}\left(d\right).$$

*By taking p=2 in *(132)*, we obtain*

(135) $$0\leq R_{G_{X}}\left(d\right)-R_{X}\;\left(d\right)\leq \frac{1}{2}\log\left(\frac{\pi e}{2}\right).$$

*The bounds in *(134)* and *(135)* tell us that the rate-distortion function of any log-concave source is approximated by that of a Gaussian source. In particular, approximating $R_{X}\;\left(d\right)$ of an arbitrary log-concave source by*

(136) $${\hat{R}}_{X}\left(d\right)=\frac{1}{2}\log\frac{\sigma^{2}}{d}-\frac{1}{4}\log\left(\frac{\pi e}{2}\right),$$

*we guarantee the approximation error $|R_{X}\;\left(d\right)-{\hat{R}}_{X}\left(d\right)|$ of at most $\frac{1}{4}\log\left(\frac{\pi e}{2}\right)\approx \frac{1}{2}$ bits.*

### 6.3. Proof of Theorem 11

**Proof.**  If $|K_{X}{|}^{\frac{1}{n}}>d$, then we choose $\hat{X}=\left(1-\frac{d}{|K_{X}{|}^{\frac{1}{n}}}\right)\left(X+Z\right)$, where $Z∼N\left(0,\frac{d}{|K_{X}{|}^{\frac{1}{n}}-d}\cdot K_{X}\right)$ is independent of *X*. This choice is admissible by independence of *X* and *Z* and the fact that $K_{X}$ and $K_{Z}$ are proportionals. Upper-bounding the rate-distortion function by the mutual information between *X* and $\hat{X}$, we have

(137) $$\begin{array}{c} R_{X}^{cov}\left(d\right)\leq h\left(X+Z\right)-h\left(Z\right)\leq \frac{n}{2}\log\frac{|K_{X}{|}^{\frac{1}{n}}}{d}. \end{array}$$

Since the Shannon lower bound for determinant constraint coincides with that for the mean-square error constraint,

(138) $$R_{X}^{cov}\left(d\right)\geq \underset{-}{R}{}_{X}\;\left(d\right)=h\left(X\right)-\frac{n}{2}\log\left(2\pi ed\right).$$

On the other hand, using (137), we have

(139) $$\begin{array}{c} R_{X}^{cov}\left(d\right)-\underset{-}{R}{}_{X}\;\left(d\right)\leq D(X||G_{X})\leq \frac{n}{2}\log\left(2\pi ec\left(n\right)\right), \end{array}$$

where (139) follows from Corollary 3.

If $|K_{X}{|}^{\frac{1}{n}}\leq d$, then we put $\hat{X}\equiv 0$, which leads to $R_{X}^{cov}\left(d\right)=0$. ☐

## 7. New Bounds on the Capacity of Memoryless Additive Channels

Recall that the capacity of such a channel is

(140) $$C_{Z}\left(P\right)=\underset{X:\frac{1}{n}{∥X∥}_{2}^{2}\leq P}{\sup}I\left(X;X+Z\right)=\underset{X:\frac{1}{n}{∥X∥}_{2}^{2}\leq P}{\sup}h\left(X+Z\right)-h(Z).$$

We compare the capacity $C_{Z}$ of a channel with log-concave additive noise with the capacity of the Gaussian channel.

### 7.1. Proof of Theorem 12

**Proof.**  The lower bound is well known, as mentioned in (19). To obtain the upper bound, we first use the upper bound on the differential entropy (1) to conclude that

(141) $$h\left(X+Z\right)\leq \frac{1}{2}\log\left(2\pi e\left(P+\mathrm{Var}\left[Z\right]\right)\right),$$

for every random variable *X* such that ${∥X∥}_{2}^{2}\leq P$. By combining (140), (141) and (32), we deduce that

(142) $$C_{Z}\left(P\right)\leq \frac{1}{2}\log\left(2\pi e\left(P+\mathrm{Var}\left[Z\right]\right)\right)-\frac{1}{2}\log\left(4\mathrm{Var}\left[Z\right]\right)=\frac{1}{2}\log\left(\frac{\pi e}{2}\left(1+\frac{P}{\mathrm{Var}[Z]}\right)\right),$$

which is the desired result. ☐

### 7.2. Proof of Theorem 13

**Proof.**  The lower bound is well known, as mentioned in (19). To obtain the upper bound, we write

(143) $$h\left(X+Z\right)-h\left(Z\right)\leq \frac{n}{2}\log\left(2\pi e|K_{X+Z}{|}^{\frac{1}{n}}\right)-h\left(Z\right)\leq \frac{n}{2}\log\left(2\pi e\;c\left(n\right)\left(\frac{\frac{1}{n}{∥Z∥}_{2}^{2}}{|K_{Z}{|}^{\frac{1}{n}}}+\frac{P}{|K_{Z}{|}^{\frac{1}{n}}}\right)\right),$$

where $c\left(n\right)=\frac{n^{2}e^{2}}{\left(n+2\right)4\sqrt{2}}$ in general, and $c\left(n\right)=\frac{e^{2}}{2}$ if *Z* is unconditional. The first inequality in (143) is obtained from the upper bound on the differential entropy (38). The last inequality in (143) is obtained by applying the arithmetic-geometric mean inequality and Theorem 4. ☐

## 8. Conclusions

Several recent results show that the entropy of log-concave probability densities have nice properties. For example, reverse, strengthened and stable versions of the entropy power inequality were recently obtained for log-concave random vectors (see e.g., [3,11,35,36,37,38]). This line of developments suggest that, in some sense, log-concave random vectors behave like Gaussians.

Our work follows this line of results, by establishing a new lower bound on differential entropy for log-concave random variables in (4), for log-concave random vectors with possibly dependent coordinates in (37), and for γ-concave random variables in (43). We made use of the new lower bounds in several applications. First, we derived reverse entropy power inequalities with explicit constants for uncorrelated, possibly dependent log-concave random vectors in (12) and (47). We also showed a universal bound on the difference between the rate-distortion function and the Shannon lower bound for log-concave random variables in Figure 1a and Figure 1b, and for log-concave random vectors in (59). Finally, we established an upper bound on the capacity of memoryless additive noise channels when the noise is a log-concave random vector in (20) and (66).

Under the Gaussian assumption, information-theoretic limits in many communication scenarios admit simple closed-form expressions. Our work demonstrates that, at least in three such scenarios (source coding, channel coding and joint source-channel coding), the information-theoretic limits admit a closed-form approximation with at most 1 bit of error if the Gaussian assumption is relaxed to the log-concave one. We hope that the approach will be useful in gaining insights into those communication and data processing scenarios in which the Gaussianity of the observed distributions is violated but the log-concavity is preserved.
